# Tunability and stability of gold nanoparticles obtained from chloroauric acid and sodium thiosulfate reaction

**DOI:** 10.1186/1556-276X-7-337

**Published:** 2012-06-22

**Authors:** Guandong Zhang, Jacek B Jasinski, Justin Lee Howell, Dhruvinkumar Patel, Dennis P Stephens, Andre M Gobin

**Affiliations:** 1 Bioengineering Department, J.B. Speed School of Engineering, Room 411, Lutz Hall, Belknap campus, Louisville, KY 40292, USA; 2 Conn Center for Renewable Energy Research, J.B. Speed School of Engineering, University of Louisville, Louisville, KY 40292, USA

**Keywords:** gold nanoparticles, gold colloid, gold nanoplates, near-infrared absorption, surface plasmon resonance, sodium thiosulfate, core-shell structure

## Abstract

In the quest for producing an effective, clinically relevant therapeutic agent, scalability, repeatability, and stability are paramount. In this paper, gold nanoparticles (GNPs) with precisely controlled near-infrared (NIR) absorption are synthesized by a single-step reaction of HAuCl_4_ and Na_2_S_2_O_3_ without assistance of additional templates, capping reagents, or seeds. The anisotropy in the shape of gold nanoparticles offers high NIR absorption, making it therapeutically relevant. The synthesized products consist of GNPs with different shapes and sizes, including small spherical colloid gold particles and non-spherical gold crystals. The NIR absorption wavelengths and particle size increase with increasing molar ratio of HAuCl_4_/Na_2_S_2_O_3_. Non-spherical gold particles can be further purified and separated by centrifugation to improve the NIR-absorbing fraction of particles. In-depth studies reveal that GNPs with good structural and optical stability only form in a certain range of the HAuCl_4_/Na_2_S_2_O_3_ molar ratio, whereas higher molar ratios result in unstable GNPs, which lose their NIR absorption peak due to decomposition and reassembly via Ostwald ripening. Tuning the optical absorption of the gold nanoparticles in the NIR regime via a robust and repeatable method will improve many applications requiring large quantities of desired NIR-absorbing nanoparticles.

## Background

Metal nanoparticles are one of the basic building blocks of nanotechnology. Gold nanoparticles (GNPs) have attracted enormous attention in chemistry, biomedicine, and electronics due to their very small size, oxide-free surfaces, bio-conjugation properties, good biocompatibility, and unique optical properties. Specifically, because of their optical activity in the near infrared (NIR), GNPs are extensively utilized in immunoassays [1,2], drug delivery systems [3] as well as imaging, detection, and thermal therapy of cancer [4-6]. These applications have sparked great interest in the development of synthetic methods for preparing different gold-based nanostructures. The anisotropy in nanoparticle shape offers high near-infrared absorption and improved Raman scattering [7]. Based on Mie scattering theory, shifts in the surface plasmon resonance (SPR) [8] occur when the particles deviate from spherical geometry. Non-spherical gold nanoparticles present multiple absorption bands correlating with their multiple axes, and they can support both propagating and localized surface plasmon resonances [7]. The number of SPR peaks usually increases as the symmetry of nanoparticles decreases; spherical nanoparticles exhibit only one peak, whereas two and three peaks are often observed in nanorods, nanodisks, and triangular nanoplates, respectively. Many anisotropic gold nanostructures like gold nanotubes [9,10], nanocages [11], gold nanoshells [6], gold nanorods [12], and gold triangular nanoprisms [13,14] have been developed and demonstrate enhanced and adjustable absorption in the NIR region. However, most of these gold nanostructures require a complicated multistep and time-consuming synthesis process, which includes shaping the particles by use of templates, kinetically controlling the facet growth rates of seeds with assistance of capping reagents, and assembly of preformed spherical colloid nanoparticles [7].

In this work, GNPs with controllable NIR absorption were synthesized by the reaction of chloroauric acid and sodium thiosulfate. This reaction was derived from the reaction of chloroauric acid and sodium sulfide that Zhou et al. first reported, whereby a proposed core-shell-type Au_2_S nanoparticle structure was produced via a two-step reduction of HAuCl_4_ by Na_2_S [15,16]. Later, Norman et.al proposed that the resulting optical properties are simply from aggregation of gold nanoparticles [15,17]. In molecular sensor studies based on scattering spectroscopy, Raschke et al. reported that gold products from Na_2_S reaction showed great improvement in scattering compared to solid GNPs, and those particles have a dielectric nanocrystal property and behave like gold nanoshells [18]. Subsequent investigations revealed that this reaction lacks reproducibility because the Na_2_S solution requires an aging process, and the aging time and reaction conditions required for this process were not well defined [19]. Schwartzberg et al. mentioned that the Na_2_S solution is not chemically stable during the aging process. Na_2_S may convert to different compounds, and sodium thiosulfate is one of the final compounds producing GNPs [20]. These findings encouraged us to attempt to reveal the key factor that dominates the nanostructure formation and the stability of the GNPs in the HAuCl_4_/Na_2_S_2_O_3_ reaction. We found that the NIR absorption of the gold products from this reaction can be well controlled and show good reproducibility when the molar ratio of HAuCl_4_/Na_2_S_2_O_3_ is in a suitable range. The instability of the GNPs is affected by the reaction conditions, resulting in the diversification of the nanostructures.

## Methods

GNPs were prepared by mixing 1.71 mM HAuCl_4_ (Au 49.50%; Alfa Aesar, Ward Hill, MA, USA) with 3 mM Na_2_S_2_O_3_ (99.999%; Aldrich, St. Louis, MO, USA) solution. The Na_2_S_2_O_3_ solution is quickly added into the HAuCl_4_ solution with the desired volume ratio and vortexed for 20 s for uniform mixing. The water used in the experiments was purified by a Thermo Scientific Easypure II system (18.2 MΩ cm; Thermo Scientific Corp., Logan, UT, USA). GNPs were purified and separated by an Allegra® X-12 Series Centrifuge (Beckman Coulter Inc., Brea, CA, USA). The as-synthesized GNP suspensions were centrifuged at 1,000 × *g* for 20 min, and then, the pellets were dispersed in deionized (DI) water for further study. The optical absorbance and intensity of nanoparticles were measured by a UV-visible-IR spectrophotometer (Cary-50Bio, Varian, Palo Alto, CA, USA). The hydrodynamic size of the nanoparticles was measured by a Zetasizer (Nano-ZS90, Malvern Instruments Ltd., Worcestershire, UK). An FEI Tecnai F20 transmission electron microscope (TEM; FEI Company, Hillsboro, OR, USA) operated at 200 KV was used to determine the shape and size of the GNPs.

## Results and discussion

### NIR absorption of the gold nanoparticles from sodium thiosulfate reaction

Gold ions are electropositive and can be reduced by various reducing agents such as borohydrate, amines, alcohols, and carboxylic acids. The most common methods use sodium citrate, sodium borohydrate, and ascorbic acid and usually produce spherical GNPs. Sodium thiosulfate, as a common reagent, has been used in many applications, such as silver recovery in photographic process and leaching of gold from mines. In the gold-leaching process, sodium thiosulfate works as a complexing agent. The reaction is complicated but can be briefly expressed as the following equation [21]:

(1) $$4\text{Au}+{8\text{S}}_{2}{\text{O}}_{3}{}^{2-}+{\text{O}}_{2}+{2\text{H}}_{2}\text{O}\to 4{\left[\text{Au}{\left({\text{S}}_{2}{\text{O}}_{3}\right)}_{2}\right]}^{3-}+{4\text{OH}}^{-}$$

Sodium thiosulfate solution is weakly alkaline and was reported as a reducing reagent both in alkaline [22] conditions for silver nanoparticle formation and in moderately acidic conditions [23] for the preparation of selenium nanoparticles, as shown in Equations 2 and 3:

(2) $${\text{S}}_{2}{\text{O}}_{3}{}^{2-}+{6\text{H}}^{-0}\to {2\text{SO}}_{3}{}^{2-}+{3\text{H}}_{2}\text{O}+{4\text{e}}^{-}\left(-0.57\text{ V}\right)$$

(3) $${\text{S}}_{2}{\text{O}}_{3}{}^{2-}+{5\text{H}}_{2}\text{O}\to {2\text{SO}}_{4}{}^{2-}+10{\text{H}}^{+}+{8\text{e}}^{-}\left(-1.5\text{ V}\right)$$

**Figure 1:**
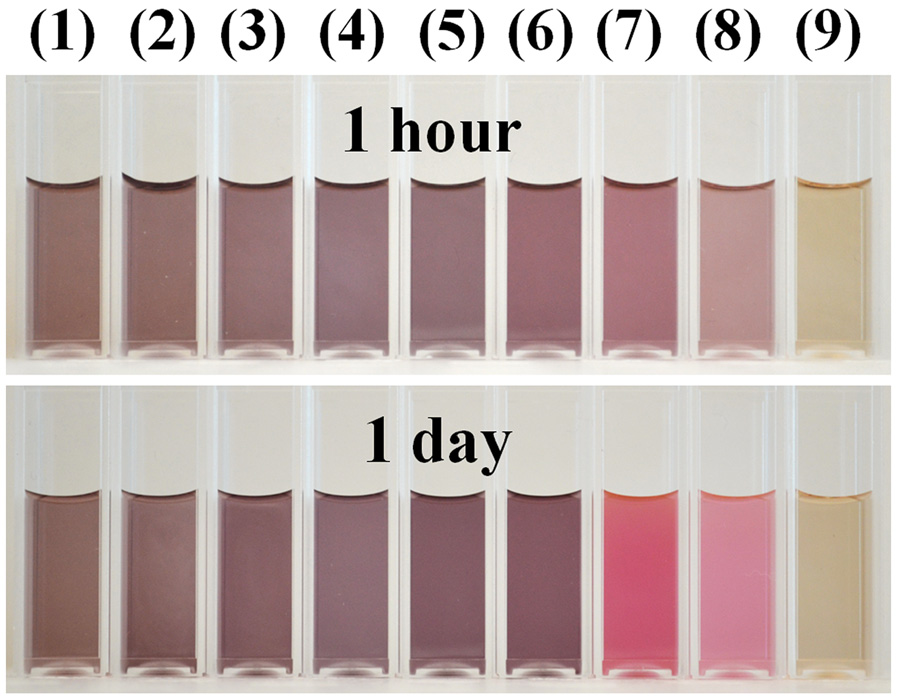
**GNP samples after mixing Na**_**2**_**S**_**2**_**O**_**3**_**and HAuCl**_**4**_**solutions.** The GNP solutions after mixing 2.2, 2.0, 1.8, 1.6, 1.4, 1.2, 1.0, 0.8, and 4.0 mL (samples 1 through 9, respectively) of 3.0 mM Na_2_S_2_O_3_ with 5 mL of 1.71 mM HAuCl_4_ at 1 h and 1 day, respectively. Photos were taken after the samples were diluted five times with water.

**Figure 2:**
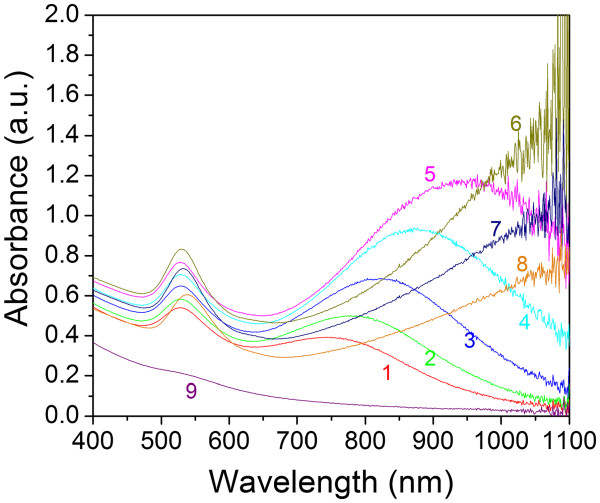
**Optical spectra of the nine GNP samples.** UV–vis-NIR spectra of the nine GNP samples measured at 1 h after mixing Na_2_S_2_O_3_ with HAuCl_4_ solution. In these measurements, all the samples were diluted five times with water.

In the reaction of HAuCl_4_ and Na_2_S_2_O_3_, sodium thiosulfate has the ability to reduce Au^3+^ to Au^0^. In order to interpret the mechanism of this reaction, nine representative samples were presented. They were synthesized by mixing 2.2, 2.0, 1.8, 1.6, 1.4, 1.2, 1.0, 0.8, and 4.0 mL (samples 1 through 9, respectively) of 3.0 mM Na_2_S_2_O_3_ with 5 mL of 1.71 mM HAuCl_4_. Within the first minute of adding Na_2_S_2_O_3_ to HAuCl_4_, all the samples showed a dark yellow color. As time progressed, the solution presented different colors. After a few minutes, the color of samples 1 to 6 turned purple, and this color was retained afterwards. Samples 7 and 8 showed the same purple color at 40 min, but after a few hours, the color had differentiated from a purple to bright brown. After dilution, these samples showed reddish to pink color when light was viewed through the sample. Sample 9 showed a quick color change to dark yellow after a few minutes and then retained this color afterwards. These colors indicate the formation of GNPs with different optical properties. Figure 1 shows the photos of these GNP samples after mixing Na_2_S_2_O_3_ and HAuCl_4_ solutions at 1 h and 1 day, respectively. To clearly distinguish the colors, the product solutions were diluted five times with water. Figure 2 shows the optical spectra of the nine GNP samples at 1 h. In samples 1 to 8, two plasmon resonance peaks are clearly observed. The first SPR peak centered at around 530 nm is the characteristic SPR of the spherical gold structures [24,25], and the second peak SPR component at the higher NIR wavelength is attributed to the multiple SPR band from the non-spherical gold nanostructures. We can see the tendency that the NIR peak wavelength increases with increasing the HAuCl_4_/Na_2_S_2_O_3_ molar ratio (samples 6 to 8, the NIR peaks are close or above 1,100 nm, beyond the measurement limit of our UV-visible (UV–vis)-NIR spectrometer). Unlike samples 1 to 8, in sample 9, where the HAuCl_4_/Na_2_S_2_O_3_ molar ratio is extremely low (approximately 0.71), only one weak SPR peak at 530 nm can be seen.

**Figure 3:**
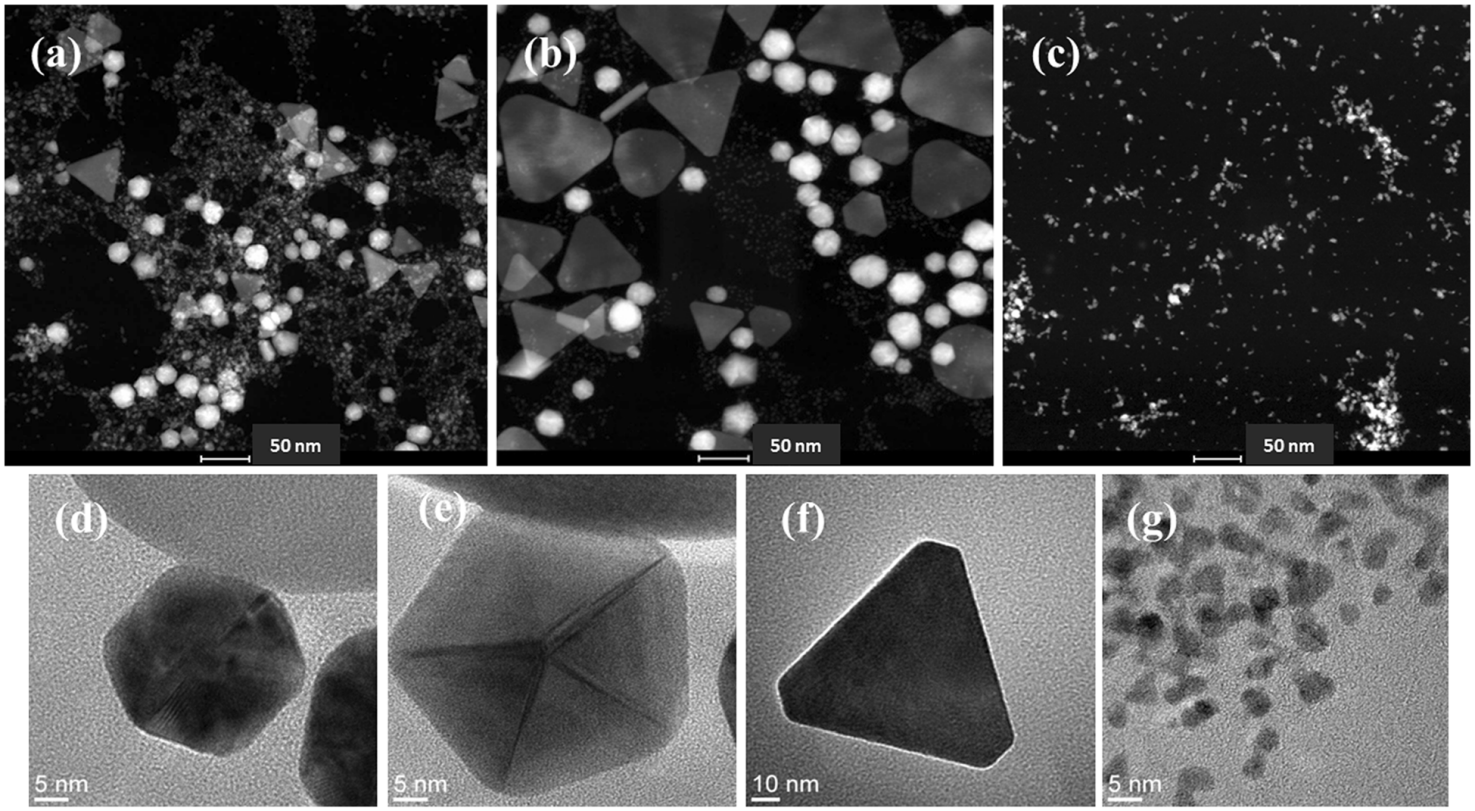
**Transmission electron microscopy images.** STEM *Z*-contrast (**a**, **b**, **c**) and HRTEM (**d**, **e**, **f**, **g**) images of the GNPs from the reaction of HAuCl_4_ and Na_2_S_2_O_3_. Top row shows Au particles synthesized by mixing 5 mL 1.71 mM HAuCl_4_ with (a) 2.2 mL 3.0 mM Na_2_S_2_O_3_ yielding a 750-nm NIR Peak, (b) 1.4 mL 3.0 mM Na_2_S_2_O_3_ yielding a 950-nm NIR absorption, and (c) 4.0 mL 3.0 mM Na_2_S_2_O_3_, which has no NIR peak and only one SPR band at 530 nm. (d, e, f, g) High-resolution TEM images of the typical species of the GNPs in the products: (d) cuboctahedron, (e) pentagon, (f) truncated triangle plate, and (g) colloidal gold particles.

**Figure 4:**
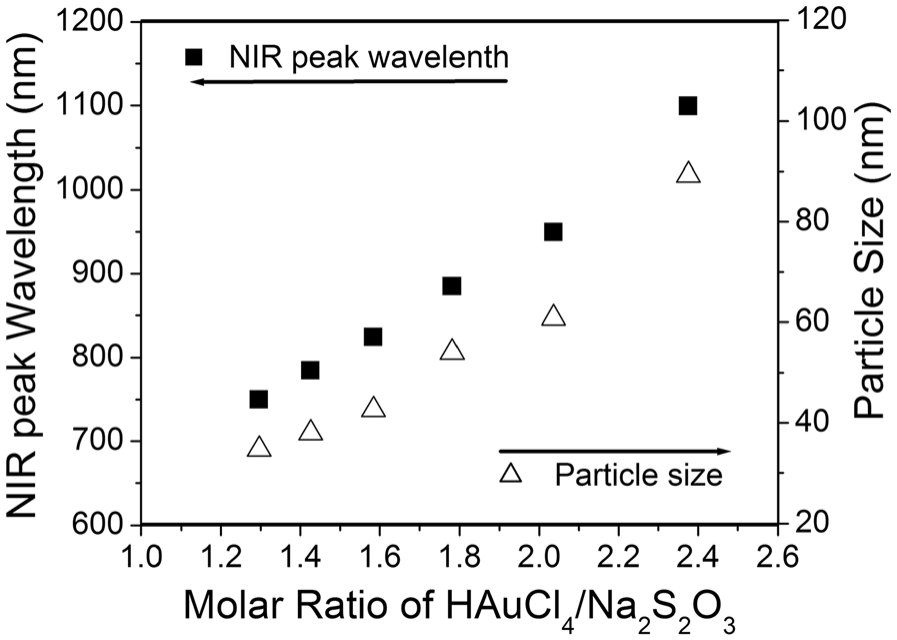
**Hydrodynamic size from dynamic light scattering measurements and the NIR peak wavelengths.** NIR peak wavelength and the hydrodynamic size of gold nanoparticles as functions of the molar ratio of HAuCl_4_/Na_2_S_2_O_3._ (only shows samples 1 to 6 with NIR peaks in the UV–vis-NIR measurable range).

Figure 3 shows the scanning transmission electron microscopy (STEM) *Z*-contrast images of three typical samples from the reaction. Figure 3a,b is the TEM image of samples 1 and 5, which has their second NIR SPR band at 750 and 950 nm, respectively. These products are the mixtures of gold particles with different shapes and sizes: the non-spherical gold crystals are interspersed among smaller colloidal GNPs. The spherical colloid GNPs are less than 5 nm. The non-spherical gold crystals contain the pseudo-spherical structures, such as truncated octahedron, pentagons, and cuboctahedron, as well as the anisotropic nanostructures with lower symmetry, including triangular and truncated triangular-shaped plate structures. Since the *Z*-contrast STEM image is the high-annular dark-field image, the intensity of the Rutherford scattered beam is directly proportional to *Z*^2^, where *Z* is the atomic number of the scattering element. The pseudo-spherical particles show higher brightness than the nanoplates in TEM images due to their higher apparent atomic number resulting from larger thickness. In Figure 3a, the pseudo-spherical crystals have diameters in the range of 15 to 30 nm, and the edges of the nanoplates are in the range of 40 to 60 nm. In Figure 3b, the size of pseudo-spherical particles increases to 30 to 45 nm, and the edge of triangular nanoplates increases to 45 to 90 nm. The thickness of the nanoplate structures, estimated from *Z*-contrast STEM images, is about 8.5 ± 1.5 nm. A rough approximation was made here that the dark field contrast changes linearly with the GNP thickness [26], and the data from a number of smaller pseudo-spherical nanoparticles were used for the calibration. The TEM image of sample 9 is shown in Figure 3c. This sample contains only spherical colloid GNPs with an average size around 3 nm, which correlates with only one SPR band at 530 nm. Figure 3d,e,f,g shows the high-resolution TEM images of the typical species of GNPs in the product: cuboctahedrons, pentagons, truncated triangle plates, and colloidal gold particles, respectively. Figure 4 shows the hydrodynamic size from dynamic light scattering (DLS) measurements and the NIR peak wavelengths as functions of the molar ratio of HAuCl_4_/Na_2_S_2_O_3_ (samples 1 to 6). In the DLS spectra, the size of small spherical colloid GNPs and the size of non-spherical gold particles can be monitored. Figure 4 shows the DLS average sizes of non-spherical gold particles, and the measurements are consistent with the TEM observation. The SPR of noble metal nanoparticles is dominated by their particle size [27] and shape [15,28]. The key feature of this synthesis method is that the optical properties of the GNPs vary with their size and the population of different non-spherical particles. It is easy to tune their NIR absorption wavelengths by adjusting reaction conditions and further separation.

### Crystal structure of the gold nanoparticles

**Figure 5:**
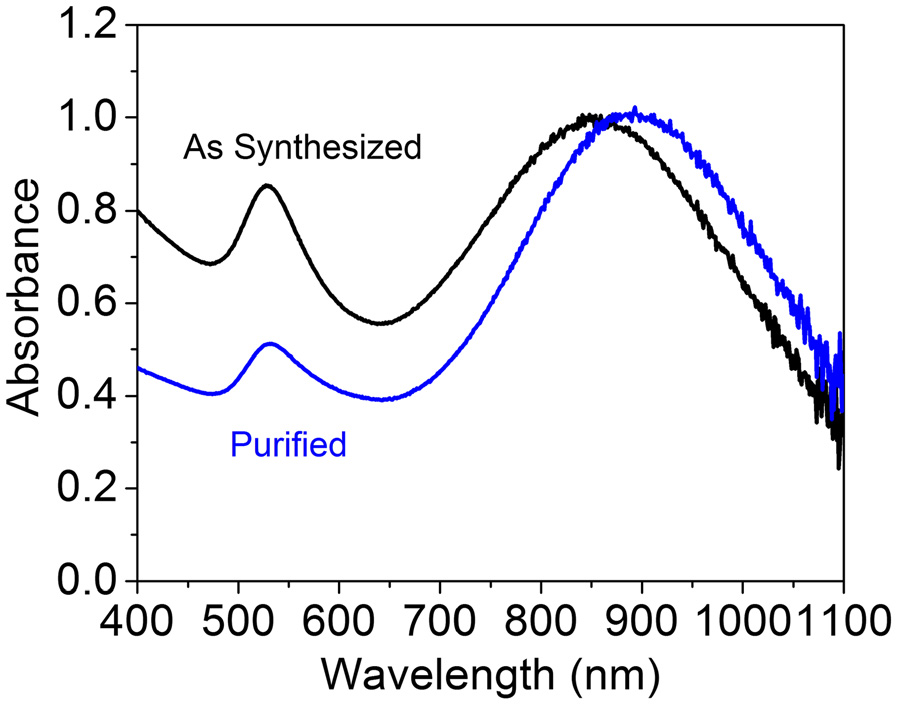
**UV–vis-NIR spectra of gold nanoparticles shown before and after purification via centrifugation.** The sample is separated at 1,000 × *g* for 20 min.

**Figure 6:**
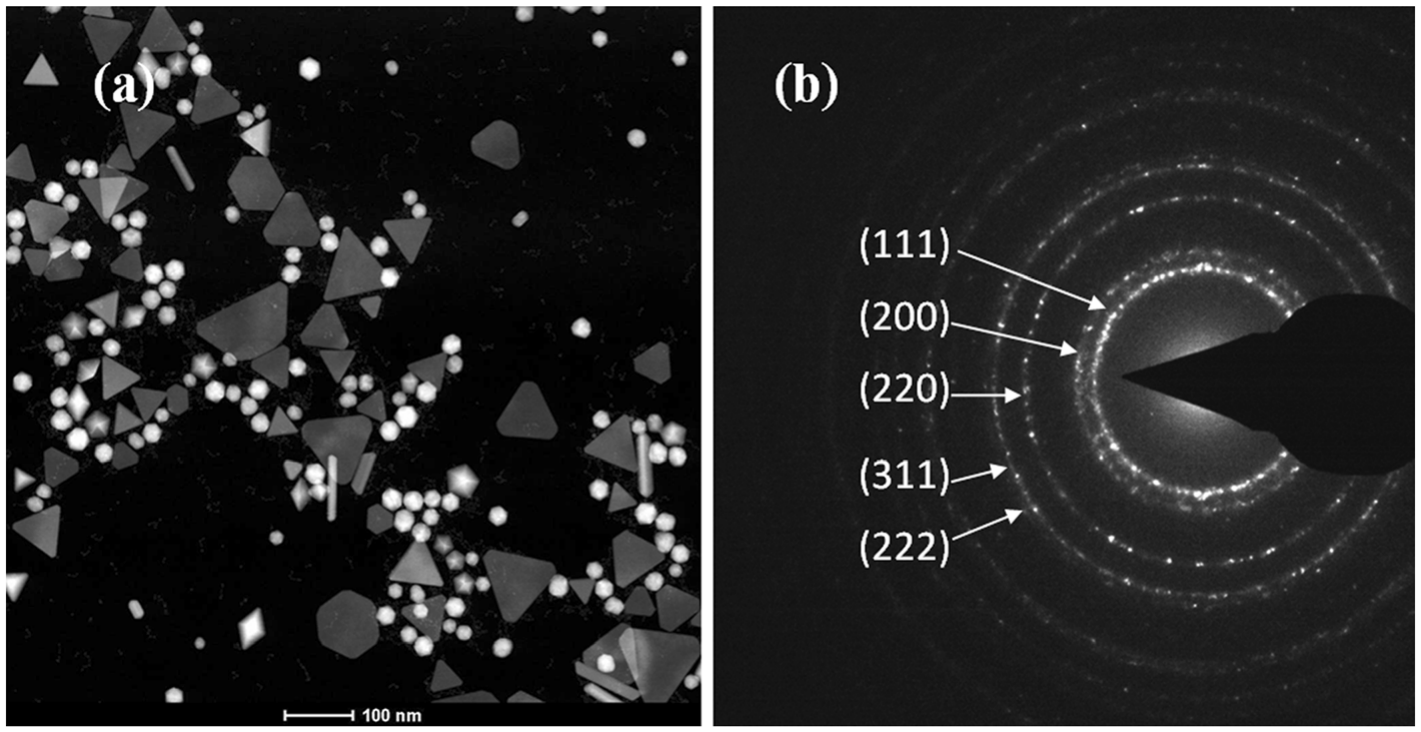
**TEM image and electron diffraction pattern of the purified gold nanoparticles.** (**a**) TEM image showing the morphology of the gold crystals. (**b**) Electron diffraction pattern from the GNPs showing the (111), (200), (220), (311), and (222) reflections of gold.

To separate the larger NIR-absorbing particles from the smaller colloidal gold particles, a centrifugation process was used to purify the synthesized products. Figure 5 shows the UV–vis-NIR spectra of GNPs before and after the centrifugation process. For comparison, samples were diluted to 1 optical density (OD). After centrifugation, the NIR peak of the GNPs shifted from 850 to 890 nm, accompanying a great decrease in the intensity of the SPR band at 530 nm. Figure 6a shows the *Z*-contrast STEM images after purification. Different species of gold particles are clearly present. Most of the small spherical colloid GNPs were removed after centrifugation. Figure 6b shows the diffraction pattern obtained from the purified GNPs, with rings corresponding to the (111), (200), (220), (311), and (222) reflections of the face-centered-cubic (fcc) structure of gold.

**Figure 7:**
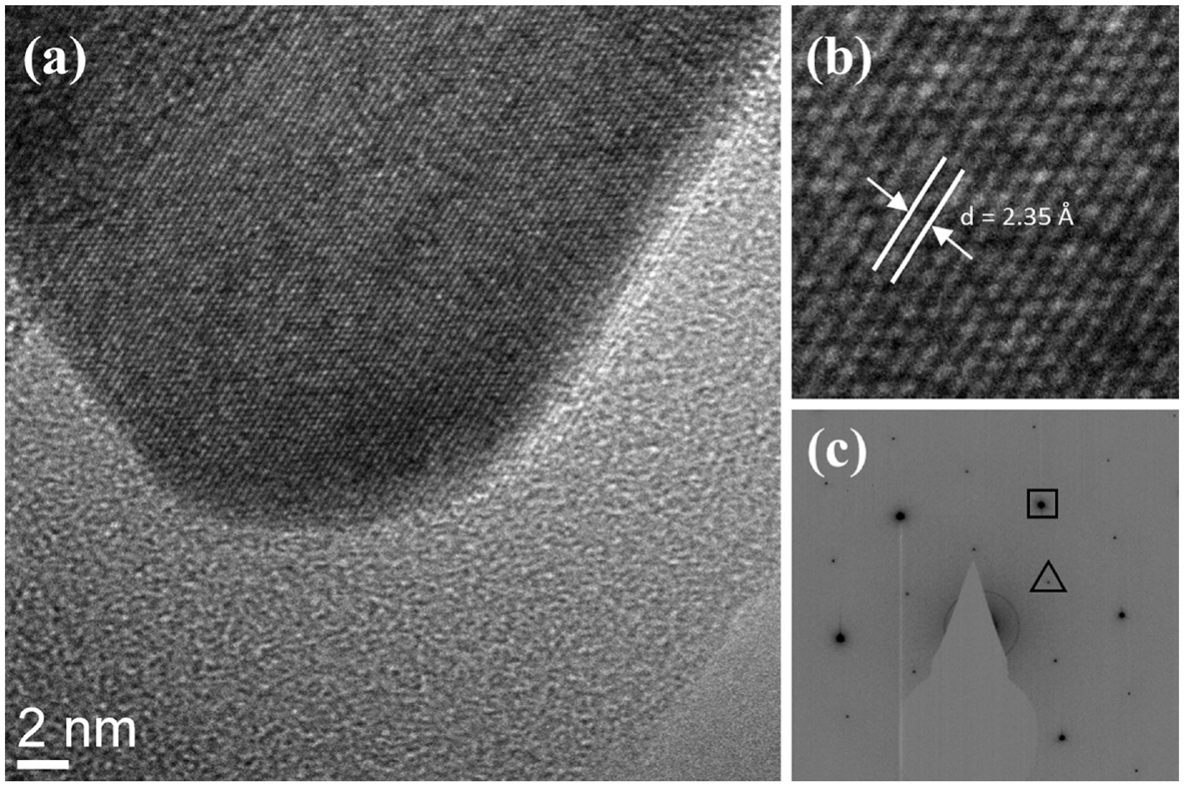
**High-resolution TEM image of the vertex of one of the nanoplate structure (a).** Its enlarged section (**b**) and SAED pattern (**c**) of the gold nanoplate structure.

Figure 7a shows a high-resolution TEM image of the vertex of one of the nanoplate structure with well-resolved (111)-type crystallographic planes (measured D-spacing of about 2.35 Å, as shown in Figure 7b), which run parallel to the long sidewalls of the structures. Figure 7c shows the selected area electron diffraction (SAED) pattern of a typical nanoplate. The pattern has a six-fold symmetry indicating the {111} zone axis, in agreement with the indexing, which can be performed self-consistently on the fcc gold structure using {220} (box-selected) and weak (1/3) {422} (triangle-selected) spots. The lattice spacing of the (220) planes measured from this pattern agrees very well with the value of 2.039 Å reported for gold. The presence of weak (1/3) {422} spots is most likely due to {111} twin planes within the nanoplates [29]. These nanoplates have the same structure as gold triangular nanoplates synthesized with the assistance of surfactants as capping agents [13,14], gold nanoplates prepared from lemongrass extract [30], and the silver nanoplate structures [31].

### Unstable gold nanoparticles and mechanism

**Figure 8:**
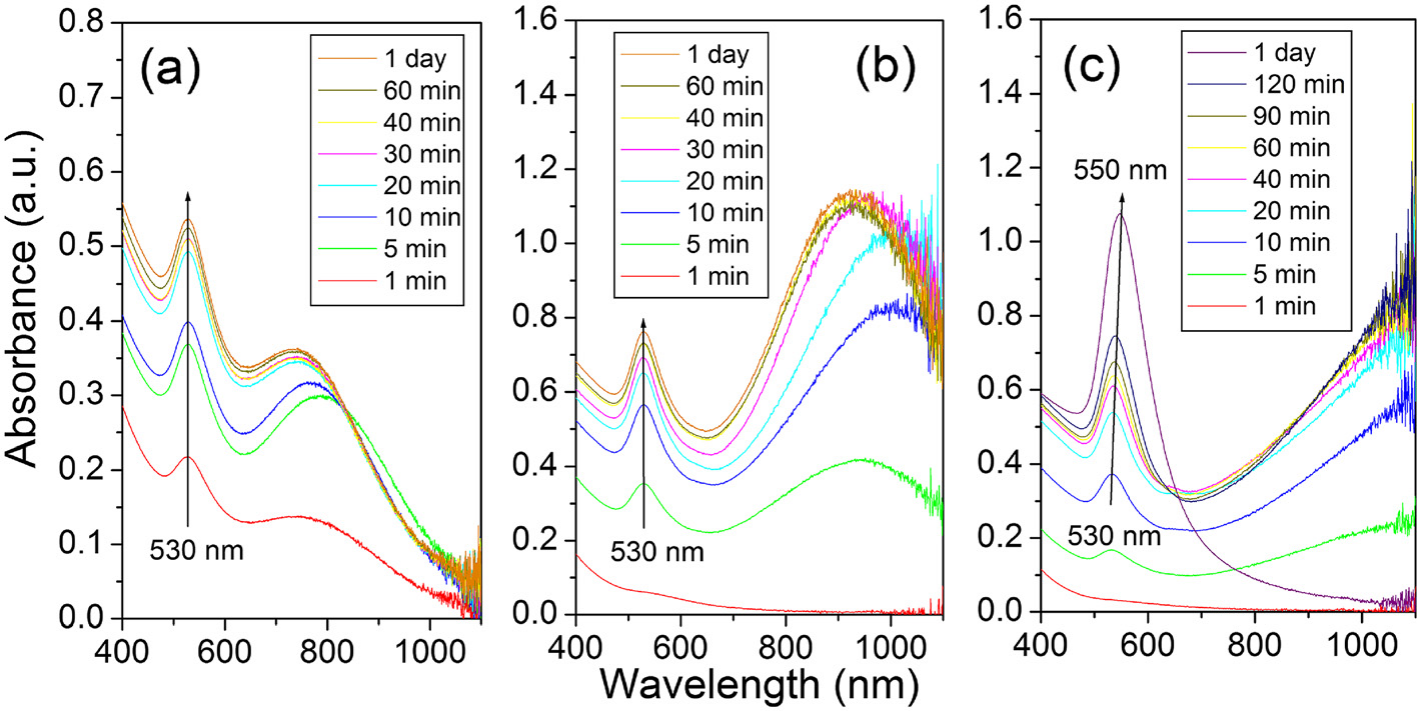
**The evolution of the optical absorption spectrum of three samples.** With different volume ratios of HAuCl_4_ and Na_2_S_2_O_3_ during the reactions: (**a**) 5 ml HAuCl_4_ + 2.2 ml Na_2_S_2_O_3_, (**b**) 5 ml HAuCl_4_ + 1.4 ml Na_2_S_2_O_3_, and (**c**) 5 ml HAuCl_4_ + 0.8 ml Na_2_S_2_O_3_. Samples were taken during the reaction and diluted five times with DI water then immediately measured by UV–vis-NIR spectrophotometer.

Optical absorption measurements indicate that the suitable range of the HAuCl_4_/Na_2_S_2_O_3_ molar ratio for producing stable non-spherical GNPs is between 1.3 and 2.0, corresponding to the particles’ NIR absorption wavelength from 750 to 950 nm. When the molar ratio is higher than 2, the gold nanostructures become unstable. Figure 8a,b,c shows the time evolution of the optical absorption spectrum of samples 1, 5, and 8 during the reaction, respectively. Samples 1 and 5 presented in Figure 8a,b show similar behavior. After 5 min, two clear peaks belonging to the transverse SPR and multiple SPR band can be clearly seen at 530 nm and at a higher NIR region. The intensity of both peaks increases while the reactions progress, and after around 40 min, the increasing of intensity becomes extremely slow, indicating that the GNPs are formed and stabilized in the early 40 min. A typical unstable sample is presented in Figure 8c, which displays the NIR peak above 1,100 nm. Unlike the stable samples, the reaction takes much longer, and both SPRs shift with time. After 1 day, the peak at 530 nm shifts to 550 nm, and the NIR SPR bands disappear.

**Figure 9:**
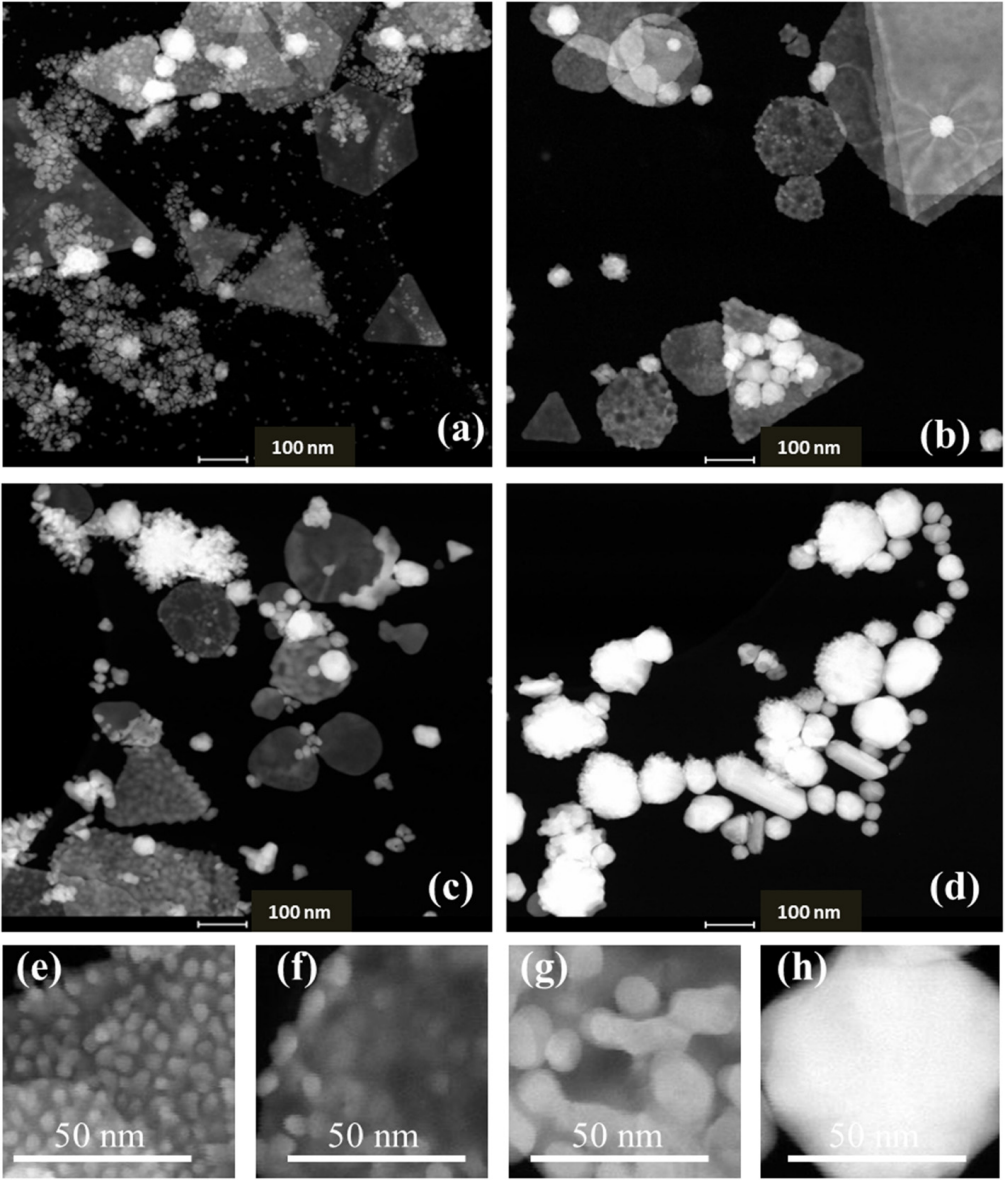
**TEM images of the growth progress of gold nanoparticles.** After mixing 5 mL 1.71 mM HAuCl_4_ with 0.8 ml of 3.0 mM Na_2_S_2_O_3_. (**a**) Product at 40 min, (**b**) 2 h, (**c**) 4 h, and (**d**) 1 day. (**e**, **f**, **g**, and **h**) show the gain sizes on the gold crystal surface of the products at 40 min, 2 h, 4 h, and 1 day, respectively.

With the assistance of TEM analysis, the evolution of the unstable particle structure and the quenching mechanism of NIR absorption can be interpreted. Figure 9 compares the morphology of sample 8, prepared by mixing 5 mL of 1.71 mM HAuCl_4_ with 0.8 mL of 3.0 mM Na_2_S_2_O_3_, after 40 min, 2 h, 4 h, and 1 day (Figure 9a,b,c,d, respectively). At 40 min, the solution contains a high density of colloid particles in addition to nanoplates and pseudo-spherical GNPs, as shown in Figure 9a. Typically, nanoparticles tend to aggregate during their synthesis, and the stabilizers, such as surfactant, small organic molecules, or polymers, play the role to protect nanoparticles after their formation through steric hindrance, thereby preventing aggregation. The Na_2_S_2_O_3_ solution is a complex system, which may contain many sulfur-based compounds. Besides the SO_4_^2−^ and SO_3_^2−^ species listed in Equations 2 and 3, the self oxidation-reduction of Na_2_S_2_O_3_ may produce trace S^2−^ and S^0^ (${\text{S}}_{2}{\text{O}}_{3}{}^{2-}\to {\text{SO}}_{4}{}^{2-}+{\text{S}}^{2-}$ or ${\text{S}}_{2}{\text{O}}_{3}{}^{2-}\to {\text{SO}}_{3}{}^{2-}+{\text{S}}^{0}$) and further convert to other sulfur derivatives [32]. Some of these species possibly function as ‘capping reagents’ or stabilizer, which direct crystal shape during the crystallization and stabilize the GNPs. For high molar ratios of HAuCl_4_ and Na_2_S_2_O_3_, the lack of capping reagents disturbs the crystallization process. Meanwhile, since the molar ratio of Cl^−^ and H^+^ ions to the freshly formed GNPs is higher, Ostwald ripening will affect these nanostructures. Ostwald ripening is a thermodynamically driven process in which smaller crystals are sacrificed by transition of atoms from the surface to the solution and then deposited onto larger crystals. This is driven by the higher surface energy of the smaller particles and the favored energy state of the larger particles. As can be seen, at 2 h and later, the colloid is already absent from the solution and is found to decorate larger GNPs (Figure 9b,c,d). The size of the colloid on the larger GNP increases with time (Figure 9e,f,g,h) through the Ostwald ripening process. Notably, 1 day after the reaction, the nanoplates disappear completely from the sample. This agrees with the total quenching of NIR absorption shown in Figure 8c and can be understood as a result of the system free energy reduction. In brief, nanoplates initially formed during the reaction represent low free energy nanostructures due to their Au (111) facets. When the colloid particles start to decorate and roughen the nanoplate surfaces, their surface energy gradually increases and eventually leads to the decomposition of nanoplates and their reassembly into less anisotropic, more spherical, and lower energy nanostructures. The Ostwald ripening process, as well as the decomposition and reassembling of larger particles, is enabled by the efficient halide-assisted transport of Au atoms. The effect takes place in an environment containing large amounts of Cl^−^ and H^+^ ions, and ionic Au-Cl complexes serve as transport species, which accelerate the gold to redeposit on some crystal surfaces [33,34]. Ostwald ripening behavior in the gold-based nanostructure interface has been reported by a number of researchers. Liang et al. [35] observed that during the SiO_2_/Au core-shell synthesis, the formation and morphology of gold nanoshell were affected by chloride-dependent Ostwald ripening. At low pH value, the gold shells reorganized to form different shaped structures. Zhao et al. [36] reported that due to intraparticle ripening, flower-shaped gold particles could be prepared by adjusting the pH and amount of chlorine ions. Lou et al. [33] reported the encapsulation and Ostwald ripening of Au and Au-Cl complexes within the Au-silica structures, in which chloride was found to be an efficient mediating ligand. Our study provides a basis to produce GNPs on a large scale in a short time. GNPs with good optical and chemical stability only formed within a narrow range in which the molar ratio of HAuCl_4_/Na_2_S_2_O_3_ ranged from 1.3 to 2.0 when the concentration of HAuCl_4_ and Na_2_S_2_O_3_ are fixed at 1.71 and 3 mM, respectively. Under the same reaction conditions (concentration, volume of the reagents), the NIR absorption peak of the GNP products can be duplicated. After purification by centrifugation, samples prepared at concentrations of 20 to 100 OD can be quickly dispersed in water with no changes in the optical absorption intensity or SPR shift even after prolonged storage at 4 °C. Tuning the optical absorption of the GNPs in the NIR region via a robust and repeatable method has great advantages reflected in the application of these GNPs in areas such as cancer therapy via photothermal ablation as well as cancer detection and imaging.

## Conclusions

In summary, we report on a convenient synthesis process to precisely control the optical absorption within the NIR region and established the suitable range of concentrations to allow stable nanoparticle formation. In this procedure, a single-step reaction of HAuCl_4_ and Na_2_S_2_O_3_ was examined in details to analyze the products of self-assembly. The nanoparticles produced from this reaction include small spherical colloidal gold particles with resonance at 530 nm and anisotropic gold nanostructures with NIR resonance. We found that the placement of the peak resonance into the NIR is controllable and repeatable with increasing molar ratios of HAuCl_4_/Na_2_S_2_O_3_. From this, it was found that in order to achieve a peak resonance above 950 nm, a molar ratio of HAuCl_4_/Na_2_S_2_O_3_ > 2.0 was required and resulted in unstable nanoparticles. The instability appears to be due to Ostwald ripening behavior based on TEM analysis over time for reactants with molar ratio greater than 2.0. Our study outlines an easy way to produce GNPs with tunable NIR absorption on a large scale in a short time and serves as the basis for additional studies to improve the efficiency of the synthesis system. This work will benefit many applications in the physical, chemical, and biomedical fields where strong NIR-absorbing nanoparticles may be used for energy transfer to create heat.

## Abbreviations

DLS: dynamic light scattering; GNPs: gold nanoparticles; NIR, near-infrared; OD: optical density; SAED: selected area electron diffraction; SPR: surface plasmon resonance; STEM: scanning transmission electron microscopy; TEM: transmission electron microscope.

## Competing interests

The authors declare that they have no competing interests.

## Authors’ contributions

GZ, JLH, and DPS carried out the synthesis of gold nanoparticles and optical measurements. GZ and JBJ completed the structural studies and characterization of gold nanoparticles. GZ and JBJ drafted the manuscript. DP participated in the interpretation of experimental data and discussions. AMG supervised the experimental design and took part in the discussion and the preparation of the manuscript. All authors read and approved the final manuscript.

## Authors’ information

GZ, JLH, DPS, DP, and AMG are from the Department of Bioengineering, J.B. Speed School of Engineering, University of Louisville. AMG is an assistant professor. GZ is a senior research associate. JLH and DPS are bachelor degree students. DP is a master degree student. JBJ is a research scientist at the Conn Center for Renewable Energy Research, J.B. Speed School of Engineering, University of Louisville.

## References

[B1] Grubisha DS Lipert RJ Park H-Y Driskell J Porter MD Femtomolar detection of prostate-specific antigen: an immunoassay based on surface-enhanced Raman scattering and immunogold labels Anal Chem 2003 75 5936 5943 14588035 10.1021/ac034356f

[B2] Hirsch LR Jackson JB Lee A Halas NJ West JL A whole blood immunoassay using gold nanoshells Anal Chem 2003 75 2377 2381 12918980 10.1021/ac0262210

[B3] Niidome T Yamagata M Okamoto Y Akiyama Y Takahashi H Kawano T Katayama Y Niidome Y PEG-modified gold nanorods with a stealth character for in vivo applications J Cont Rel 2006 114 343 347 10.1016/j.jconrel.2006.06.017 16876898

[B4] Alric C Taleb J Le Duc G Mandon C Billotey C Le Meur-Herland A Brochard T Vocanson F Janier M Perriat P Roux S Tillement O Gadolinium chelate coated gold nanoparticles as contrast agents for both X-ray computed tomography and magnetic resonance imaging J Am Chem Soc 2008 130 5908 5915 18407638 10.1021/ja078176p

[B5] Astruc D Daniel MC Ruiz J Dendrimers and gold nanoparticles as exo-receptors sensing biologically important anions Chem Commun (Camb) 2004 23 2637 2649 15568050 10.1039/b410399h

[B6] Gobin AM Lee MH Halas NJ James WD Drezek RA West JL Near-infrared resonant nanoshells for combined optical imaging and photothermal cancer therapy Nano Lett 2007 7 1929 1934 17550297 10.1021/nl070610y

[B7] Tréguer-Delapierre M Majimel J Mornet S Duguet E Ravaine S Synthesis of non-spherical gold nanoparticles Gold Bulletin 2008 41 195 207

[B8] Feldheim DL Foss CA Metal Nanoparticles: Synthesis, Characterization and Applications 2002 Marcel Dekker, Inc., New York

[B9] Sun Y Xia Y Shape-controlled synthesis of gold and silver nanoparticles Science 2002 298 2176 2179 12481134 10.1126/science.1077229

[B10] Sun Y Xia Y Gold and silver nanoparticles: a class of chromophores with colors tunable in the range from 400 to 750 nm Analyst 2003 128 686 691 12866889 10.1039/b212437h

[B11] Hu M Petrova H Chen JY McLellan JM Siekkinen AR Marquez M Li XD Xia YN Hartland GV Ultrafast laser studies of the photothermal properties of gold nanocages J Phys Chem B 2006 110 1520 1524 16471708 10.1021/jp0571628

[B12] Huang XH El-Sayed IH Qian W El-Sayed MA Cancer cell imaging and photothermal therapy in the near-infrared region by using gold nanorods J Am Chem Soc 2006 128 2115 2120 16464114 10.1021/ja057254a

[B13] Millstone JE Park S Shuford KL Qin LD Schatz GC Mirkin CA Observation of a quadrupole plasmon mode for a colloidal solution of gold nanoprisms J Am Chem Soc 2005 127 5312 5313 15826156 10.1021/ja043245a

[B14] Chu HC Kuo CH Huang MH Thermal aqueous solution approach for the synthesis of triangular and hexagonal gold nanoplates with three different size ranges Inorg Chem 2006 45 808 813 16411718 10.1021/ic051758s

[B15] Mohr F Gold Chemistry: Applications and Future Directions in the Life Sciences 2009 WILEY-VCH, Verlag GmbH & Co. KGaA, Weinheim

[B16] Zhou HS Honma I Komiyama H Haus JW Controlled synthesis and quantum-size effect in gold-coated nanoparticles Phys Rev B 1994 50 12052 10.1103/physrevb.50.12052 9975346

[B17] Norman TJ Grant CD Magana D Zhang JZ Liu J Cao D Bridges F Van Buuren A Near infrared optical absorption of gold nanoparticle aggregates J Phys Chem B 2002 106 7005 7012

[B18] Raschke G Brogl S Susha AS Rogach AL Klar TA Feldmann J Fieres B Petkov N Bein T Nichtl A Kuerzinger K Gold nanoshells improve single nanoparticle molecular sensors Nano Lett 2004 4 1853 1857

[B19] Zhang JZ Schwartzberg AM Norman T Grant CD Liu J Bridges F Van Buuren T Comment on “gold nanoshells improve single nanoparticle molecular sensors” Nano Lett 2005 5 809 810 15826133 10.1021/nl0479379

[B20] Schwartzberg AM Grant CD van Buuren T Zhang JZ Reduction of HAuCl4 by Na2S revisited: the case for Au nanoparticle aggregates and against Au2S/Au core/shell particles J of Phy Chem C 2007 111 8892 8901

[B21] Breuer PL Jeffrey MI Thiosulfate leaching kinetics of gold in the presence of copper and ammonia Miner Eng 2000 13 1071 1081

[B22] Prochazka M Vlckova B Stepanek J Turpin PY Probing of porphyrin surface chemistry in systems with laser-ablated Ag nanoparticle hydrosol: role of thiosulfate anions Langmuir 2005 21 2956 2962 15779971 10.1021/la047307m

[B23] Lin ZH Wang CRC Evidence on the size-dependent absorption spectral evolution of selenium nanoparticles Mater Chem Phys 2005 92 591 594

[B24] Brioude A Jiang XC Pileni MP Optical properties of gold nanorods: DDA simulations supported by experiments J Phys Chem B Condens Matter Mater Surf Interfaces Biophys 2005 109 13138 13142 16852635 10.1021/jp0507288

[B25] Chen HM Peng HC Liu RS Asakura K Lee CL Lee JF Hu SF Controlling the length and shape of gold nanorods J Phys Chem B Condens Matter Mater Surf Interfaces Biophys 2005 109 19553 19555 16853528 10.1021/jp053657l

[B26] Langmore JSWaMI JP The collection of scattered electrons in dark field electron microscopy I. Elastic scattering. Optik 1973 38 335 350

[B27] Link S El-Sayed MA Size and temperature dependence of the plasmon absorption of colloidal gold nanoparticles J Phys Chem B 1999 103 4212 4217

[B28] Kelly KL Coronado E Zhao LL Schatz GC The optical properties of metal nanoparticles: the influence of size, shape, and dielectric environment J Phys Chem B 2003 107 668 677

[B29] Kirkland AI Jefferson DA Duff DG Edwards PP Gameson I Johnson BFG Smith DJ Structural studies of trigonal lamellar particles of gold and silver P Roy Soc Lond A Mat 1993 440 589 609

[B30] Shankar SS Rai A Ahmad A Sastry M Controlling the optical properties of lemongrass extract synthesized gold nanotriangles and potential application in infrared-absorbing optical coatings Chem Mater 2005 17 566 572

[B31] Jiang X Zeng Q Yu A A self-seeding coreduction method for shape control of silver nanoplates Nanotechnology 2006 17 4929 4935

[B32] Chen JY Yang SZ Ke JJ Mao MH Studies and Developments of Hydrometallurgy 1998 Metallurgical Industry Press, Beijing

[B33] Lou XW Yuan CL Rhoades E Zhang Q Archer LA Encapsulation and Ostwald ripening of Au and Au-Cl complex nanostructures in silica shells Adv Funct Mater 2006 16 1679 1684

[B34] Cheng WL Dong SJ Wang EK Iodine-induced gold-nanoparticle fusion/fragmentation/aggregation and iodine-linked nanostructured assemblies on a glass substrate Angew Chem Int Edit 2003 42 449 452 10.1002/anie.200390136 12569514

[B35] Liang Z Liu Y Ng S Li X Lai L Luo S Liu S The effect of pH value on the formation of gold nanoshells J of Nanopart Res 2011 13 1 11

[B36] Zhao LL Ji XH Sun XJ Li J Yang WS Peng XG Formation and stability of gold nanoflowers by the seeding approach: the effect of intraparticle ripening J Phys Chem C 2009 113 16645 16651

